# Hypothalamic PVN CRH Neurons Signal Through PVN GABA Neurons to Suppress GnRH Pulse Generator Frequency in Female Mice

**DOI:** 10.1210/endocr/bqad075

**Published:** 2023-05-29

**Authors:** Caitlin McIntyre, Xiao Feng Li, Deyana Ivanova, Jun Wang, Kevin T O’Byrne

**Affiliations:** Department of Women and Children's Health, Faculty of Life Sciences and Medicine, King's College London, Guy's Campus, SE1 1UL, UK; Department of Women and Children's Health, Faculty of Life Sciences and Medicine, King's College London, Guy's Campus, SE1 1UL, UK; Department of Women and Children's Health, Faculty of Life Sciences and Medicine, King's College London, Guy's Campus, SE1 1UL, UK; Reproductive Medicine Center, Affiliated Hospital of Guizhou Medical University, Guizhou 550004, China; Department of Women and Children's Health, Faculty of Life Sciences and Medicine, King's College London, Guy's Campus, SE1 1UL, UK

**Keywords:** PVN, CRH, GABA, LH, stress

## Abstract

Corticotropin-releasing hormone (CRH) neurons in the paraventricular nucleus of the hypothalamus (PVN) are central to the stress response. Chemogenetic activation of PVN CRH neurons decreases LH pulse frequency but the mechanism is unknown. In the present study, optogenetic stimulation of PVN CRH neurons suppressed LH pulse frequency in estradiol-replaced ovariectomized CRH-cre mice, and this effect was augmented or attenuated by intra-PVN GABA_A_ or GABA_B_ receptor antagonism, respectively. PVN CRH neurons signal to local GABA neurons, which may provide a possible indirect mechanism by which PVN CRH neurons suppress LH pulse frequency. Optogenetic stimulation of potential PVN GABAergic projection terminals in the hypothalamic arcuate nucleus in ovariectomized estradiol-replaced Vgat-cre-tdTomato mice via an optic fiber implanted in the arcuate nucleus suppressed LH pulse frequency. To further determine whether PVN CRH neurons signal through PVN GABA neurons to suppress LH pulsatility, we combined recombinase mice with intersectional vectors to selectively target these neurons. CRH-cre::Vgat-FlpO mice expressing the stimulatory opsin ChRmine in non-GABAergic CRH neurons alone or in combination with the inhibitory opsin NpHR3.3 in non-CRH-expressing GABA neurons in the PVN were used. Optogenetic stimulation of non-GABAergic CRH neurons suppressed pulsatile LH secretion; however, LH pulse frequency was not affected when CRH neurons were stimulated and PVN GABA neurons were simultaneously inhibited. Together, these studies demonstrate that suppression of LH pulse frequency in response to PVN CRH neuronal activation is mediated by GABAergic signalling intrinsic to the PVN and may incorporate PVN GABAergic projection to the hypothalamic GnRH pulse generator.

Both the hypothalamus-pituitary-adrenal (HPA) and hypothalamus-pituitary-gonadal axes are intimately connected. LH secretion is regulated by the pulsatile release of GnRH. The GnRH pulse generator is primarily composed of a subpopulation of neurons in the arcuate nucleus (ARC) of the hypothalamus that coexpress kisspeptin, neurokinin B, and dynorphin A and are therefore referred to as kisspeptin/neurokinin B/dynorphin (KNDy) neurons. Acute stress has been shown to decrease KNDy neuronal activity and suppress pulsatile LH secretion (1, 2). Increased HPA axis activity is strongly linked to impaired reproductive function (3, 4); administration of chronic corticosterone (CORT) disrupts estrous cyclicity in mice (5) and inhibits pulsatile LH secretion in estrogen-replaced ovariectomized (OVX) mice (6).

A major population of corticotrophin-releasing hormone (CRH) neurons, located in the parvocellular division of the paraventricular nucleus (PVN) of the hypothalamus, play an essential role in regulating HPA axis activation and therefore CORT secretion, particularly in response to ultradian and diurnal rhythms (7) and stress exposure (8, 9). PVN CRH neurons are tightly regulated by GABAergic inputs from the amygdala and peri-PVN zone surrounding the PVN, as well as locally from within the PVN (10). The balance between inhibitory and stimulatory inputs to PVN CRH neurons modulates HPA axis activation, and acute stress has been shown to decrease GABAergic and increase glutamatergic transmission to PVN CRH neurons (11-14). GABAergic transmission mediates phasic and tonic inhibition of PVN CRH neurons through activation of GABA_A_ receptors (GABA_A_R) at synaptic and extrasynaptic sites, respectively (15). Conversely, GABA has been shown to feedback to suppress GABA release at presynaptic terminals through GABA_B_R activation (15).

Although early lesioning studies suggested the PVN was not critical in mediating stress-induced suppression of LH secretion (16), growing evidence highlights PVN CRH neurons as modulators of LH pulse frequency. The PVN has been identified as a mediator of metabolic stress-induced suppression of reproductive function (17, 18) and estrogen (E_2_) action at the level of the PVN has been shown to play a permissive role in enabling LH pulse suppression in fasting OVX rats (19). CRH expression is increased in the PVN in murine models of functional hypothalamic amenorrhea (20) and selective chemogenetic activation of PVN CRH neurons has been shown to suppress LH pulse frequency in mice (21). However, the mechanism through which PVN CRH neurons mediate LH pulse suppression remains unclear.

The PVN provides a rich source of primary afferents to ARC KNDy neurons (22), and the latter express CRH receptors (23). Close appositions between CRH terminals, including projections from the PVN, and ARC kisspeptin neurons have been identified (21, 23). Although these data might suggest a direct functional CRH projection from the PVN to the GnRH pulse generator, electrophysiological recordings from kisspeptin neurons in vitro from diestrous mice show that application of CRH does not alter their firing rate, and optogenetic stimulation of PVN CRH terminals in the ARC failed to alter kisspeptin neuron firing (21), providing strong support that PVN CRH neurons indirectly regulate the GnRH pulse generator. Given the significant proportion of primary efferents from the PVN to the KNDy neurons and the findings that these projections do not express CRH (22), CRH neurons may be signalling to other local PVN neurons, which in turn project to ARC KNDy neurons to indirectly suppress LH pulse frequency.

In the present study, we first examined the effects of optogenetic activation of PVN CRH neurons in conjunction with local GABA receptor antagonism on LH pulse frequency in OVX mice with and without E_2_ replacement. To further explore the neural circuitry in the PVN modulating GnRH pulse generator activity, we used an optogenetic approach to selectively stimulate potential PVN GABAergic projection terminals in the ARC to determine the effect on LH pulse frequency. Next, we applied intersectional strategies that used the Cre/LoxP and Flp/FRT to selectively target heterogenous PVN neuronal populations based on multiple genetic features. To determine if CRH neurons are signalling through PVN GABA neurons to suppress GnRH pulse generator activity, we selectively stimulated PVN CRH neurons while simultaneously inhibiting PVN GABA neurons optogenetically and examined the effects on LH pulse frequency and additionally the CORT response as a measure of HPA axis activation.

## Materials and Methods

### Animals

All mice were kept under standard conditions with a 12:12 hours light-dark cycle at 25 ± 1 °C with ad libitum access to standard chow and water. Female mice weighing between 19 and 21 g and aged 6 to 8 weeks were used. The King's College London Animal Welfare and Ethical Review Body approved all animal procedures performed. Procedures were in accordance with UK home office regulation.

Adult CRH-cre mice homozygous for the allele CRH-cre (Jax stock #012704, B6(Cg)-Crh^tm1(cre)Zjh^/J, Jackson Laboratory, Bar Harbor, ME, USA) or Vgat-cre mice homozygous for the allele Slc32a1tm2(cre)Lowl (Jax stock #028862, B6J.129S6(FVB)-Slc32a1^tm2(cre)lowl^/MwarJ; Jackson Laboratory) were crossbred in house with adult homozygous tm9(CAG-tdTomato)Hz mice (Jax stock #007909, B6.Cg-Gt(ROSA)26Sortm9(CAG-tdTomato)Hze/J, Jackson Laboratory) to acquire double heterozygous CRH-cre-tdTomato or Vgat-cre-TdTomato mice with the tomato transgene being expressed upon cre-mediated recombination in CRH-cre or Vgat-cre expressing cells.

For experiments using intersectional optogenetic techniques, homozygous CRH-cre mice were crossbred with heterozygous Vgat-FlpO mice (Jax stock: #029591, B6.Cg-Slc32a1^tm1.1(flpo)Hze^/J, Jackson Laboratory) to acquire double heterozygous CRH-cre::Vgat-FlpO combinational line.

Mice were genotyped using PCR to confirm applicable heterozygosity for either CRH-cre (primers 5′-3′: common, 10574, CTTACACATTTCGTCCTAGCC; wild-type forward, 10575, CACGACCAGGCTGCGGCTAAC; mutant forward, 105756, CAATGTATCTTATCATGTCTGGATCC), tdtomato (primers 5′-3′: wild-type forward oIMR9020—AAGGGAGCTGCAGTGGAGTA; wild-type reverse oIMR9021—CCGAAAATCTGTGGGAAGTC; mutant reverse WPRE oIMR9103—GGCATTAAAGCAGCGTATCC; mutant forward tdTomato oIMR9105—CTGTTCCTGTACGGCATGG), Vgat-cre (primers 5′-3′: common, 12 785—CTTCGTCATCGGCGGCATCTG; wild-type reverse 12 786—CAGGGCGATGTGGAATAGAAA; mutant reverse oIMR8292—CCAAAAGACGGCAATATGGT); and Vgat-FlpO (primers 5′-3′: wild-type forward, 31 174—GTCTGCGTTTCTGTCGTCCT; wild-type reverse, 31 175—CTCAAGGTCAAGTTTCCAAGC; mutant reverse, 19 775—TGCATCGCATTGTCTGAGTAG; mutant forward, 30707- GACAGCCGTGAACAGAAGG).

### Stereotaxic Injection of Viral Constructs and Implantation Fiber Optic or Optofluid Cannula

All surgical procedures were carried out under general anesthesia using ketamine (Vetalar, 100 mg/kg, IP; Pfizer, Sandwich, UK) and xylazine (Rompun, 10 mg/kg, IP; Bayer, Leverkusen, Germany) under aseptic conditions. Mice were secured in a David Kopf stereotaxic frame (Kopf Instruments, Model 900) and either bilaterally OVX or OVX and subcutaneously implanted with a silastic capsule containing 17-β E_2_ dissolved in peanut oil to a concentration of 36 μg/mL and filled in 1.4-cm capsules or inner and outer diameter: 1.575/3.175 mm as previously described (24). These capsules provide low levels of circulating E_2_ found in the diestrous phase of the cycle. To reveal the skull, a midline incision was made in the scalp and 2 small bone screws were inserted into the skull and a small hole was drilled above the position of the PVN. Coordinates for the right PVN (0.2 mm lateral, 0.82 mm from bregma, at a depth of 4.7 mm below the skull surface) were obtained from the mouse brain atlas of Paxinos and Franklin (25). Viral constructs were unilateral injected over 10 minutes into the PVN, using a 2-μL Hamilton microsyringe (Esslab, Essex, UK) attached to a robotic stereotaxic system (Neurostar, Tubingen, Germany). The needle was left in position for a further 5 minutes and then removed slowly over 2 minutes.

In experiments optogenetically activating CRH cell bodies in the PVN with or without neuropharmacological manipulation, 2 experimental groups were used: OVX only (n = 4) and OVX E_2_-replaced (n = 10) CRH-cre mice that received unilateral intra-PVN stereotaxic viral injection of the stimulatory AAV-ChR2-EYFP viral construct (300 nL AAV-EF1a-double floxed-hChR2(H134R)-EYFP-WPRE-HGHpA, ≥ 1.8 × 10¹³ GC/mL; Serotype:9; Addgene viral prep: #20298-AAV9; RRID:Addgene_20298; gift from Karl Diesseroth). For the control group, OVX E_2_-replaced CRH-cre mice (n = 4) received a unilateral intra-PVN injection of control virus containing only the fluorescent tag EYFP (AAV-Ef1a-DIO-EYFP, 2.2 × 10^13^ GC/mL, Serotype:9; Addgene viral prep: #27056, RRID:Addgene_27056, gift from Karl Diesseroth, Addgene). A unilateral fiber optic cannula (Doric Lenses, Quebec, Canada) or Optofluid cannula (Doric Lenses) was then inserted into the PVN 0.2 mm above the injection site. For the OVX-only group injected with AAV-ChR2, all 4 mice were implanted with a fiber optic cannula. For the OVX E_2_-replaced group injected with AAV-ChR2, 4 mice were implanted with a fiber optic cannula and the remaining 6 mice were implanted with an Optofluid cannula. For the control OVX E_2_-replaced group injected with AAV-EYFP, all 4 mice were implanted with an Optofluid cannula. Once in position, the fiber optic or Optofluid cannula was secured on the skull using dental cement (Super-Bond Universal Kit, Prestige Dental, UK) and the scalp incision closed with suture.

In experiments where PVN GABAergic projection terminals in the ARC were optically stimulated, Vgat-cre-tdTomato mice received a viral injection into the PVN, but the fiber optic cannula was implanted in the ARC. These Vgat-cre-Tdtomato mice received a unilateral intra-PVN stereotaxic viral injection of either the stimulatory AAV-ChR2-EYFP viral construct (n = 7, 300 nL AAV-EF1a-double floxed-hChR2(H134R)-EYFP-WPRE-HGHpA, ≥ 1.8 × 10¹³ GC/mL; Serotype:9; Addgene viral prep: #20298-AAV9; RRID:Addgene_20298; gift from Karl Diesseroth) or a control virus containing only the fluorescent tag EYFP (n = 5, AAV-Ef1a-DIO-EYFP, 2.2 × 10^13^ GC/mL, Serotype:9; Addgene viral prep: #27056, RRID:Addgene_27056, gift from Karl Diesseroth, Addgene). To allow for the selective stimulation of PVN GABA terminals in the ARC, a fiber optic cannula (Doric Lenses) was positioned in the ARC (0.3 mm lateral, 1.2 mm posterior to bregma, and at a depth of 6.0 mm) and secured in place as described previously.

To selectively target heterogenous PVN neuronal populations based on dual genetic features, we used a combinatorial CRH-cre::Vgat-FlpO mouse model and exclusion-based intersectional viral vectors. The intersectional viral vectors used in the present study enables the conditional expression of the stimulatory opsin, ChRmine in cells that express CRH-cre but not Vgat-FlpO and, conversely, the inhibitory opsin NPHR3.3, in cells that express Vgat-FlpO but not CRH-cre. We had 2 experimental groups consisting of CRH stimulation only and CRH stimulation with simultaneous GABA inhibition and a control virus group. For the CRH stimulation-only group, CRH-cre::Vgat-FlpO OVX E_2_-replaced mice (n = 6) received a unilateral intra-PVN stereotaxic intersectional viral injection of the stimulatory AAV-Con/Foff-ChRmine-oScarlet viral construct (pAAV-nEF-Con/Foff 2.0-Chrmine-oScarlet; ≥ 1 × 10¹³ GC/mL; Serotype:8; Addgene viral prep: #137161-AAV8, RRID:Addgene_137161, gift from Karl Deisseroth and INTRSECT 2.0 Project, Addgene (26)). The Con/Foff expression cassette in this viral vector enables ChRmine expression in neurons dependent on the presence and absence of cre and FlpO, respectively; therefore, only non-GABAergic CRH neurons will be optogenetically targeted. For the CRH stimulation with simultaneous GABA inhibition group, CRH-cre::Vgat-FlpO OVX E_2_-replaced mice (n = 8) received a unilateral intra-PVN stereotaxic injection of an intersectional viral mixture containing the stimulatory AAV-Con/Foff-ChRmine-oScarlet viral construct (as previously) and the inhibitory construct AAV-Coff/Fon-NpHR3.3-EYFP (AAV-Coff/Fon-NpHR3.3-EYFP; ≥ 1 × 10¹³ GC/mL; Serotype:8; Addgene viral prep: #137161-AAV8, RRID:Addgene_137161, gift from Karl Deisseroth and INTRSECT 2.0 Project, Addgene (26)).The Coff/Fon expression cassette in this viral vector induces NpHR3.3 expression in cells dependent on the absence of cre and the presence of FlpO. Consequently, this group will express ChRmine in CRH-cre only cells and NpHR3.3 in Vgat-FlpO only cells allowing for the simultaneous optogenetic stimulation and inhibition of heterogenous CRH and GABA neurons respectively. For the control virus group, CRH-cre::Vgat-FlpO OVX E_2_ replaced mice (n = 3) received a unilateral intra-PVN injection of an viral mixture containing a cre-dependent control viral construct (AAV-Ef1a-DIO-EYFP, 2.2 × 10^13^ GC/mL, Serotype:9; Addgene viral prep: #27056, RRID:Addgene_27056, gift from Karl Diesseroth, Addgene) and a FlpO-dependent control viral construct (AAV-Ef1a-fDIO-mCherry, 2.2 × 10^13^ GC/mL, Serotype:9; Addgene viral prep: #114471-AAV9; RRID:Addgene_114471, gift from Karl Diesseroth, Addgene). This control virus mixture will result in the expression of the fluorescent tags eYFP and mCherry in CRH and GABA neurons, respectively. For all 3 groups, a fiber optic cannula (Doric Lenses) was then inserted into the PVN 0.2 mm above the injection site to allow for optogenetic manipulation as described previously. Mice were left to recover for 1 week; after the recovery period, mice were handled daily to acclimatize to experimental procedures for a further 3 weeks.

### Intra-PVN Administration of Bicuculline or CGP-35348 During Optogenetic Stimulation of PVN CRH Neurons

To determine the effect of optical stimulation of PVN CRH neurons on pulsatile LH secretion in the presence and absence of E_2_, OVX (n = 4) or OVX E_2_-replaced (n = 4) CRH-cre mice expressing AAV-ChR2 in the PVN were used. The ferrule of the fiber optic cannula was attached to a multimode fiber optic rotatory joint patch cable (Thorlabs LTD, Ely, UK) via a ceramic mating sleeve that allows mice to freely move while receiving blue light (473 nm wavelength). Laser (DPSS laser, Laserglow Technologies, Toronto, Canada) intensity was set to 10 mW at the tip of the fiber optic patch cable. The frequency and pattern of optical stimulation was controlled by software designed in house. After 1 hour of controlled blood sampling (5 μL every 5 minutes using tail-tip method (27)) without optical stimulation to determine baseline LH pulse frequency, mice received high-frequency patterned optical stimulation at 20 Hz with 10-ms pulse width and a stimulation pattern of 5 seconds on 5 seconds off (28) for 1 hour while blood sampling continued.

For neuropharmacological manipulation of GABA receptor signalling in the PVN, OVX E_2_-replaced CRH-cre mice expressing AAV-ChR2 receiving intra-PVN infusion of GABA receptor antagonists with or without optical stimulation, were connected to the laser via the ferrule of the Optofluid cannula as described previously for the fiber optic cannula, but additionally an injection cannula connected to tubing preloaded with drug solutions was inserted into the guide cannula of the Optofluid implant. The tubing for drug infusion extended beyond the cage and the distal ends were attached to a 10-μL Hamilton syringe (Waters Ltd, Elstress, UK) fitted to a PHD 2000 programmable syringe pump (Harvard Apparatus, MA, USA), allowing for a continuous infusion of the drug at a constant rate; mice were kept in the cage throughout the experiment, freely moving with food and water ad libitum. After 50 minutes of control blood sampling to determine baseline LH pulse frequency, mice were administered an initial 0.3-μL bolus injection of either the GABA_A_R antagonist bicuculline (BIC, 20.0 pmol, bicuculline, Sigma-Aldrich; n = 6), GABA_B_R antagonist CGP-35348 (CGP, 4.5 nmol, CGP-35348, Sigma-Aldrich; n = 6) or artificial cerebrospinal fluid (aCSF; n = 6) as vehicle control delivered at a rate of 0.03 μL/min over 10 minutes. The laser was turned on at 60 minutes with the same stimulation parameters as described previously and either BIC (68 pmol), CGP (15 nmol), or aCSF was continuously infused in 0.8 μL at a rate of 0.013 μL/min for the remaining 60 minutes of blood sampling. In the absence of optical stimulation, the same neuropharmacological regimens were applied and included an aCSF control group. As an additional control group, OVX E_2_-replaced CRH-cre mice (n = 4) expressing the control viral construct, AAV-Ef1a-DIO-EYFP, were subjected to tail-tip blood sampling for LH measurement while infused with aCSF and optically stimulated as described previously.

To determine the effect of optical stimulation of PVN CRH neurons on CORT secretion in the absence and presence of E_2_, OVX (n = 4) or OVX E_2_-replaced (n = 4) CRH-cre mice expressing AAV-ChR2 in PVN CRH neurons were used on a separate occasion. For CORT measurements, 15-µL blood samples were collected and stored in tubes containing 5 µL of heparinized saline (50 IU/mL). Optogenetic stimulation was initiated, as described previously, 30 minutes into the experiment (time point, 0 minutes) and lasted for 1 hour (time point 60 minutes). Blood samples were collected at −30, 0, +15, +30, and +60 minutes. As a control, OVX E_2_-replaced (n = 3) CRH-cre mice expressing AAV-ChR2 in PVN CRH neurons were subjected to tail-tip blood sampling for CORT measurement but not optogenetically stimulated. At the end of the experiment, blood samples were centrifuged at 1300 RPM for 20 minutes and plasma stored at −20 °C. Experiments were conducted over 4 to 6 weeks. All experiments were performed between 9:00 and 12:00 am. Mice received all treatments in a random order, with at least 2 but typically 4 days between experiments.

### Optogenetic Stimulation of PVN GABAergic Projection Terminals in the ARC

OVX E_2_-replaced Vgat-cre-tdTomato mice transfected with AAV-ChR2 (n = 7) or control virus (n = 5) in the PVN, were implanted with a fiber optic cannula in the ARC to allow for the selective photostimulation of potential PVN GABAergic neuron projection terminals in the ARC. Mice were connected to a blue light laser (473 nm wavelength) as described previously. Laser intensity was set to 10 mW at the tip of the fiber optic patch cable. After 1 hour of controlled blood sampling (5 μL every 5 minutes using tail-tip method (27)) without optical stimulation to determine baseline LH pulse frequency, mice received high-frequency optical stimulation at 20 Hz, with a 10-ms pulse width and a stimulation pattern of 5 seconds on 5 seconds off for 1 hour while blood sampling continued. As an additional control, mice expressing AAV-ChR2 (n = 3) were subjected to tail-tip blood sampling for LH measurement, but not optogenetically stimulated. All experiments were performed between 9:00 and 12:00 am. Mice received all treatments in a random order, with at least 3 but typically 5 days between experiments.

### Optogenetic Manipulation of CRH and GABA Neurons in the PVN

OVX E_2_-replaced CRH-cre::Vgat-FlpO mice expressing either AAV-ChRmine alone (n = 6, CRH stimulation only), AAV-ChRmine combined with AAV-NpHR3.3 (n = 8, CRH stimulation and GABA inhibition), or a control virus (n = 3, virus mixture resulting in expression of the fluorescent tags eYFP and mCherry in CRH and GABA neurons, respectively), in the PVN were connected to a green-light laser (532 nm wavelength, DPSS laser, Laserglow Technologies) as described previously. Laser intensity was set to 10 mW at the tip of the fiber optic patch cable. In the CRH stimulation-only group, optical stimulation results in the activation of non-GABAergic CRH neurons in the PVN. In the CRH stimulation and GABA inhibition group, optical stimulation results in the activation of non-GABAergic CRH neurons and the simultaneous inhibition of GABA but not CRH-expressing neurons in the PVN. ChRmine and NpHR3.3 have overlapping activation wavelength spectra (29-31) and both have previously been shown to be activated by 532 nm light enabling the simultaneous activation of both opsins (32, 33). For all 3 groups, mice underwent 1-hour tail-tip blood sampling every 5 minutes as described previously, in the absence of optical stimulation to determine baseline LH pulse frequency, and then received optical stimulation at 20 Hz with a 10-ms pulse width and a stimulation pattern of 5 seconds on 5 seconds off for 1 hour while serial blood sampling continued. As an additional control, and to complement the nonoptically stimulated OVX E_2_-replaced CRH-cre mice expressing AAV-ChR2 in the PVN described previously, the OVX E_2_-replaced CRH-cre::Vgat-FlpO mice expressing AAV-ChRmine alone (n = 3) were also bled every 5 minutes for 2 hours for LH pulse detection in the absence of optical stimulation.

To determine whether selective stimulation of PVN CRH neurons while simultaneously inhibiting PVN GABA neurons also affects CORT secretion differently from activation of PVN CRH neurons alone, OVX E_2_-replaced CRH-cre::Vgat-FlpO mice expressing AAV-ChRmine with AAV-NpHR3.3 (n = 6, CRH stimulation and GABA inhibition) or AAV-ChRmine alone (n = 6, CRH stimulation only) in the PVN were connected to a green-light laser (532 nm wave-length, DPSS laser, Laserglow Technologies) as described previously. Optogenetic stimulation was initiated, as described, 30 minutes into the experiment (time point, 0 minutes) and lasted for 1 hour (time point, 60 minutes). Blood samples for CORT measurement were collected at −30, 0, +15, +30, and +60 minutes as described previously. As a control, OVX E_2_-replaced CRH-cre::Vgat-FlpO mice expressing the control virus (n = 3, virus mixture resulting in expression of the fluorescent tags eYFP and mCherry in CRH and GABA neurons, respectively) were also bled for CORT measurement and optically stimulated in the same manner. All experiments were performed between 9:00 and 12:00 am. Mice received all treatments in a random order, with at least 3 but typically 5 days between experiments.

### Validation of AAV Injection Site and Cannula Position

After experimentation had concluded, mice received an IP lethal injection of ketamine and were transcardially perfused with heparinized saline for 5 minutes followed by phosphate-buffered (pH 7.4) 4% paraformaldehyde for 10 minutes using a pump (Minipuls; Gilson, Dunstable, UK). Brains were placed into a 15% sucrose-4% paraformaldehyde solution. After sinking, brains were transferred into a 30% sucrose-0.2 m PBS solution and stored overnight at 4 °C. Brains were snap frozen in isopropanol on dry ice and stored at −80 °C, until coronally sectioned (30 µm) using a cryostat (Bright Instrument Co., Huntingdon, UK), when every third section was collected throughout the PVN region between −1.34 and −2.70 from bregma. Sections were airdried on microscope slides and cover slipped with ProLong Anti-fade mounting medium (Molecular Probes Inc., Eugene, OR, USA). Verification of correct AAV injection site and optic fiber placement was performed using an Axioskop 2 plus microscope using AXIOVISION, version 4.7 (Carl Zeiss, Oberkochen, Germany). Images were taken using Axioskop 2 Plus microscope (Carl Zeiss).

For optogenetic experiments stimulating AAV-ChR2-EYFP in CRH-cre-tdTomato and Vgat-cre-tdTomato mice, we determined whether PVN CRH or GABA neurons, respectively, were successfully infected by merging td-Tomato fluorescence expressed in CRH or vgat cells with EYFP fluorescence in the PVN. The number of EYFP positive neurons colocalized with tdTomato fluorescence in the PVN of each animal was determined using 2 sections and the average number of neurons presented is per section per PVN. The group mean percent of colocalization was calculated by taking the average number of EYFP-positive neurons out of the average number of neurons expressing tdTomato fluorescence per 2 sections and presented as mean ± SEM %. For experiments stimulating AAV-ChR2 in CRH-cre-tdTomato mice, only data from animals with both accurate AAV injection and cannula placement in the PVN were included in the analysis. In experiments optically stimulating AAV-ChR2 expressing PVN GABA terminals in the ARC, only mice with accurate AAV injection into the PVN, and correct cannula placement in the ARC was included in the analysis. Moreover, the presence of AAV-ChR2-EYFP expressing terminals in the ARC was visually confirmed.

For experiments in CRH-cre::Vgat-FlpO mice expressing AAV-Con/Foff-ChRmine-oScarlet the number of oScarlet positive neurons in the PVN of each animal was determined using 2 sections and the average number of neurons presented is per section per PVN. In experiments where CRH-cre::Vgat-FlpO were induced to express both AAV-Con/Foff-ChRmine-oScarlet and AAV-Coff/Fon-NpHR3.3-EYFP, the number of EYFP-positive neurons and oScarlet-positive neurons in the PVN of each animal was determined using 2 sections and the average number of neurons presented is per section per PVN. Only data from animals with both accurate AAV injection and cannula placement in the PVN were included in the analysis.

### Blood Sampling Procedure and LH and CORT Measurements

Following a 1-week recovery period from surgery, mice were handled daily for 3 weeks before blood sample collection to minimize handling stress during serial blood sampling. The tail tip was excised using a sterile scalpel, and mice were left to habituate in a clean cage for 1 hour. For LH measurement, 5 μL of blood was collected every 5 minutes for 2 hours from freely moving animals (27). Samples were diluted in 45 μL of 0.2% BSA-0.05% PBS with Tween-20 (0.2% BSA and 0.05% PBST) and immediately placed on dry ice and stored at −80 °C until later analysis. Blood samples were processed using an ultrasensitive LH ELISA, as reported previously (34), using a capture antibody (monoclonal antibody, antibovine LHβ subunit, AB_2665514, UC Regents, CA, USA), mouse LH standard (AFP-5306A, dilution 1:25, provided by Albert F. Parlow, National Hormone and Pituitary Program, Torrance, CA, USA), primary antibody (polyclonal antibody, rabbit LH antiserum, AB_2665533, Harbor-UCLA, CA, USA), secondary antibody (horseradish peroxidase-linked donkey anti-rabbit IgG polyclonal antibody, AB_772206, VWR International, Leicestershire, UK). Intra-assay and interassay variations were 3.8% and 10.7%, respectively, and the functional assay sensitivity was 0.31 ng/mL. LH pulses were determined using the DynPeak algorithm (35). Average LH inter-pulse interval (IPI) (the time between 2 LH pulse peaks) was calculated for the 1-hour control period and 1-hour treatment period.

Blood samples (15 μL) collected for CORT measurement were processed using a commercially available enzyme immunoassay kit (sheep polyclonal antibody-specific for corticosterone, RBID:AB_2877626; DetectX Corticosterone Enzyme Immunoassay Kit, K014; Arbor Assays, MI, USA) with an assay sensitivity of 20.9 pg/mL and performed in accordance with the manufacturer's protocol and as previously described (36).

### Statistics

Mice were compared between groups using a 2-way ANOVA (time and treatment) and post hoc Tukey test. All statistics were performed using SigmaPlot, version 14.5 (Systat software Inc.). Data are presented as mean ± SEM and *P* < .05 was considered significant.

## Results

### Validation of AAV Injection Site and Cannula Position

**Figure 1.**
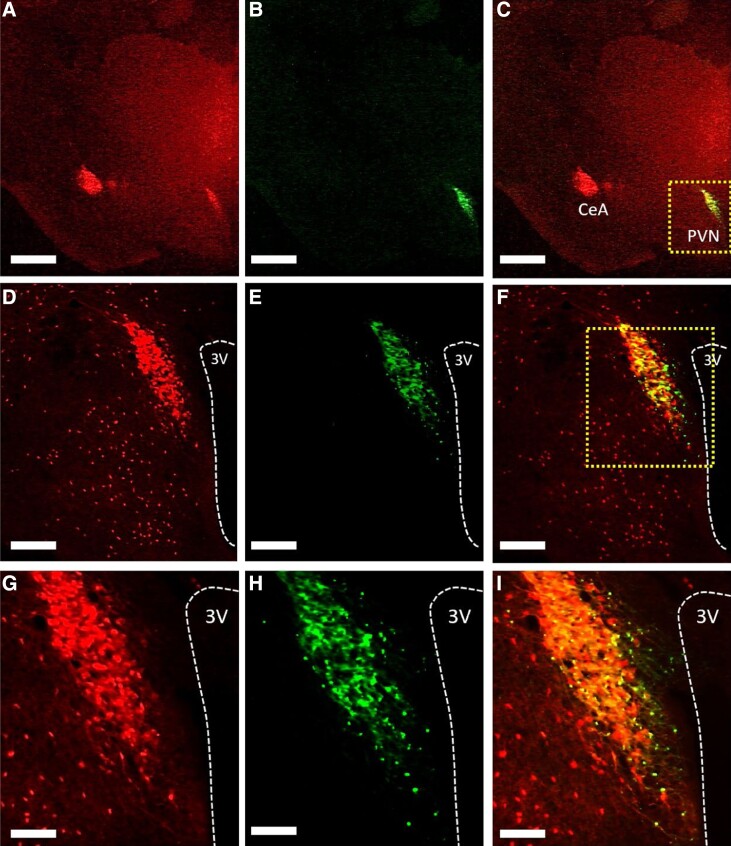
Expression of AAV-ChR2-EYFP in corticotropin-releasing hormone (CRH) neurons in the hypothalamic paraventricular nucleus (PVN). (A-I), Representative photomicrographs of dual fluorescence in CRH-cre-TdTomato neurons in the PVN of female ovariectomized (OVX) estrogen (E_2_)-replaced mice injected with AAV-ChR2-EYFP. PVN CRH neurons labeled with (A, D, G) tdTomato, (B, E, H) AAV-ChR2-EYFP, and (C, F, I) merged. Area in box in (C) and (F) shown at higher magnification in (D-F) and (G-I) respectively. Scale bars represent (A-C) 1000 µm, (D-F) 200 µm, and (G-I) 100 µm. 3 V, third ventricle (white dashed line); CeA, central amygdala.

The AAV-ChR2 viral construct used to infect CRH-cre-tdTomato or Vgat-cre-tdTomato positive neurons in the PVN was tagged with the fluorescent tag EYFP to allow for visualization under a microscope. For optogenetic stimulation of PVN CRH neurons, in OVX-only and OVX E_2_-replaced female CRH-cre-tdTomato mice, analysis of images collected from coronal sections show that mice had successful stereotaxic injection of AAV-ChR2 in the PVN and optic fiber placement in the PVN. In OVX E_2_-replaced female CRH-cre-tdTomato mice injected with AAV-ChR2 in the PVN, 4 of 4 mice and 6 of 6 mice had successful stereotaxic injection of AAV-ChR2 in the PVN and optic fiber or Optofluid placement in the PVN respectively. In OVX E_2_-replaced female CRH-cre-tdTomato mice injected with a control viral construct in the PVN 4 of 4 mice had successful stereotaxic injection of AAV-EYFP in the PVN and Optofluid cannula placement in the PVN. A representative example of AAV-ChR2 expression in a section containing the PVN showing colocalization with CRH-cre-tdTomato is illustrated in Fig. 1A-1I. The mean ± SEM number of EYFP positive cells in the PVN was 62.83 ± 5.76 per section (Fig. 1B, 1E, and 1H). Evaluation of AAV-ChR2-EYFP expression in CRH-cre-tdTomato-labeled neurons in the PVN showed that 79.61 ± 2.29% of PVN CRH-cre-tdTomato neurons coexpressed AAV-ChR2-EYFP (Fig. 1C, 1F, and 1I). Nonspecific expression of ChR2 in the PVN (EYFP single labeling) was observed in less than 3% of EYFP expressing non-tdTomato-expressing neurons in the PVN per section in all mice.

**Figure 2.**
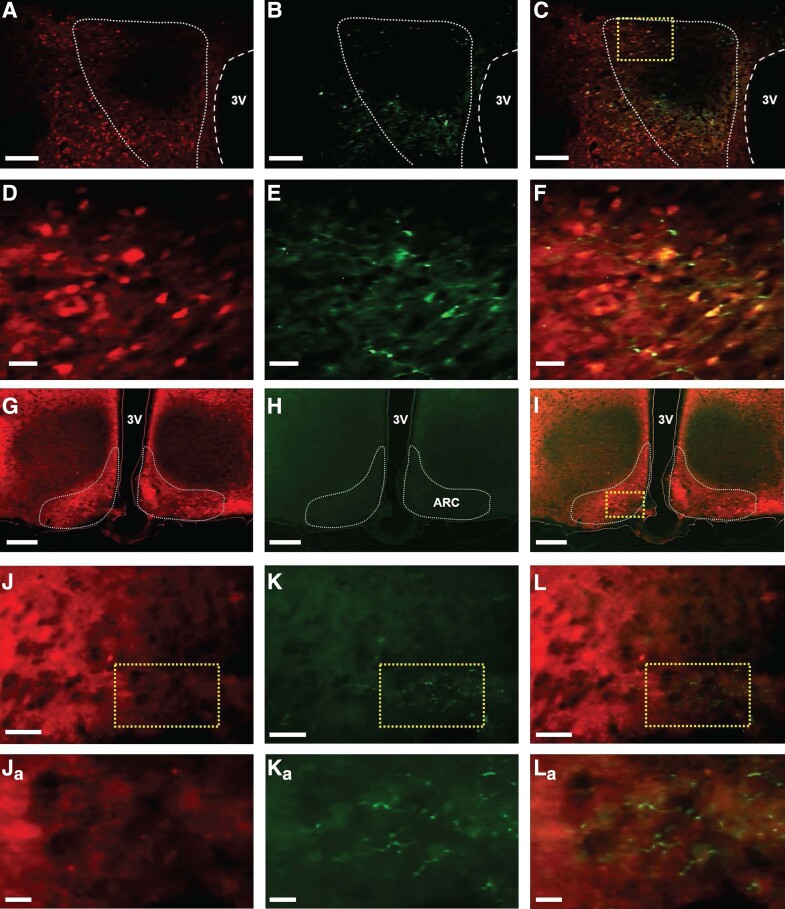
Expression of AAV-ChR2-EYFP in GABA neurons in the hypothalamic paraventricular nucleus (PVN) and identification of PVN GABA terminals in the hypothalamic arcuate nucleus (ARC). (A-F) Representative photomicrographs of dual fluorescence in Vgat-cre-tdTomato neurons in the PVN and (G-L), ARC of female ovariectomized (OVX) estrogen (E_2_)-replaced mice injected with AAV-ChR2-EYFP in the PVN. GABA neurons labeled with (A, D) tdTomato, (B, E) EYFP, and (C, F) merged in the PVN. Area in box in (C) shown at a higher magnification in (D-F). (G-L), Representative photomicrographs of the ARC showing (G, J) GABA cell bodies labeled with TdTomato, (H, K) PVN GABA projection terminals expressing ChR2-EYFP in the ARC, and (I, L) merged. (J-L) High-power view of areas (dashed box) in (I). (Ja-La), higher magnification of areas (dashed box) highlighted in (J-L), respectively, showing examples of PVN GABA terminals in the ARC. Scale bars represent (A-C) 100 µm, (G-H) 200 µm, (D-F) and (J-L) 20 µm, and (Ja-La) 10 µm. Areas outlined in white dashed line in (A-C) and (G-I) indicate the boundaries of the PVN and ARC, respectively. 3 V, the third ventricle.

For optogenetic stimulation of PVN GABA neuronal terminals in the ARC of OVX E2-replaced female mice Vgat-cre-tdTomato, analysis of images collected from coronal sections show that 6 of 7 AAV-ChR2-injected mice and 4 of 5 control virus-injected mice had successful stereotaxic injection of viral construct and optic fiber placement in the ARC. Shown are representative examples of tdTomato expression (Fig. 2A and 2D), ChR2 (Fig. 2B and 2E), and colocalization of ChR2 and tdTomato (Fig. 2C and 2F) in a section of the PVN. The mean ± SEM number of EYFP-positive cells in the PVN was 32.67 ± 3.84 per section (Fig. 2B and 2E). Evaluation of AAV-ChR2-EYFP expression in Vgat-cre-tdTomato neurons in the PVN showed 57.06 ± 4.74% of Vgat-cre-tdTomato neurons coexpressed EYFP and therefore ChR2. Nonspecific expression of ChR2 in the PVN (EYFP single labeling) was observed in less than 8% of EYFP expressing non-TdTomato-expressing neurons per section per PVN (mean ± SEM). ChR2-EYFP expressing terminals were visualized in the ARC; representative examples are illustrated in Fig. 2K and 2Ka and Fig. 2L and 2La. The phenotype of ARC neurons that ChR2-EYFP-expressing fibers potentially communicate with was not determined.

**Figure 3.**
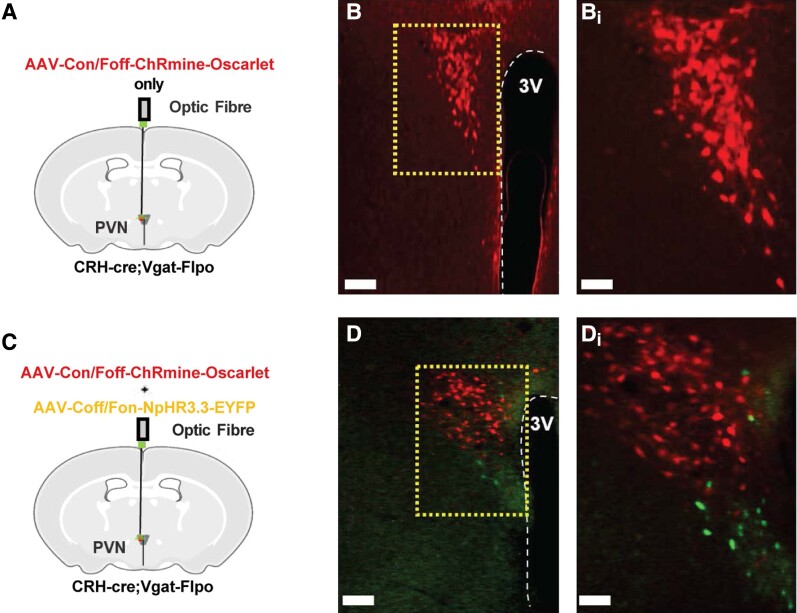
Expression of exclusion-based intersectional viral constructs AAV-Con/Foff-ChRmine-oScarlet in non-GABAergic corticotropin-releasing hormone (CRH) neurons and AAV-Coff/Fon-NpHR3.3-EYFP in non-CRH GABA neurons in the hypothalamic paraventricular nucleus (PVN). CRH-cre::Vgat-FlpO ovariectomized (OVX) estradiol (E_2_)-replaced female mice received an intra-PVN injection of (A) AAV-Con/Foff-ChRmine-oScarlet only to selectively stimulate non-GABAergic CRH neurons or (C) AAV-Con/Foff-ChRmine-oScarlet and AAV-Coff/Fon-NpHR3.3-EYFP to selectively stimulate non-GABAergic CRH neurons while simultaneously inhibiting non-CRH GABA neurons. (B and D) Representative photomicrographs of dual fluorescence of oScarlet (red) and EYFP (green) in CRH-cre::Vgat-FlpO neurons in the PVN of female OVX E_2_-replaced mice injected with either (B) AAV-Con/Foff-ChRmine-oScarlet only or (D) both AAV-Con/Foff-ChRmine-oScarlet and AAV-Coff/Fon-NpHR3.3-EYFP. (Bi, Di) Areas in dashed box shown in (B, D) at a higher power, respectively. Scale bars represent (B, D) 100 µm and (Bi, Di) 50 µm.

For optogenetic stimulation of PVN CRH neurons with or without simultaneous inhibition of PVN GABA neurons in CRH-cre::Vgat-FlpO OVX E_2_-replaced female mice, analysis of images collected from coronal sections show that 6 of 6 mice injected with AAV-Con/Foff-ChRmine-oScarlet only and 6 of 8 mice injected with AAV-Con/Foff-ChRmine-oScarlet and AAV-Coff/Fon-NpHR3.3-EYFP had both successful stereotaxic injection of AAV and optic fiber placement in the PVN. For the control virus group, 3 of 3 mice had successful injection of the control viral mixture and optic fiber positioning in the PVN. A representative example of AAV-Con/Foff-ChRmine-oScarlet expression in mice that were transduced to express AAV-ChRmine only is shown in Fig. 3B and 3Bi. The mean ± SEM number of oScarlet-positive cells in the PVN was 69.83 ± 7.07 per section (Fig. 3B and 3Bi) and no EYFP expression was visualized in mice injected with AAV-ChRmine only. A representative example of AAV-Con/Foff-ChRmine-oScarlet and AAV-Coff/Fon-NpHR3.3-EYFP expression in mice that were transduced to express both AAV-ChRmine and AAV-NpHR3.3 is shown in Fig. 3D and 3Di. The mean ± SEM number of oScarlet- and EYFP-positive cells in the PVN was 67.60 ± 14.21 and 28.2 ± 5.61 per section, respectively. No significant difference was observed in AAV-ChRmine-oScarlet expression between groups expressing either AAV-Con/Foff-ChRmine-oScarlet only or in combination with AAV-Coff/Fon-NpHR3.3-EYFP (*P* > .05). No colocalization of AAV-Con/Foff-ChRmine-oScarlet and AAV-Coff/Fon-NpHR3.3-EYFP were observed in mice that received injections of both viral constructs.

### Selective Optogenetic Stimulation of PVN CRH Neurons Suppresses LH Pulse Frequency in OVX E_2_-replaced Mice

**Figure 4.**
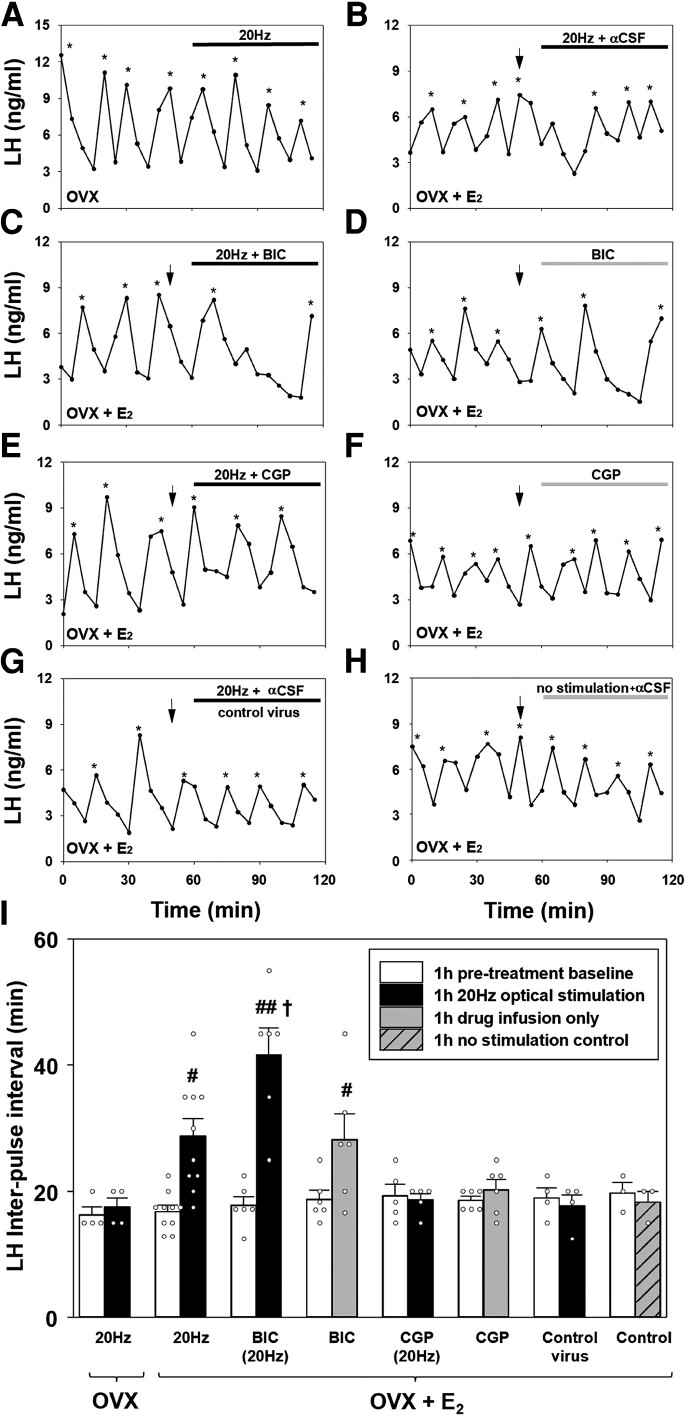
Optogenetic stimulation of corticotrophin-releasing hormone (CRH) neurons in the hypothalamic paraventricular nucleus (PVN) with blue light (473 nm) at 20 Hz (10 mW, 5 seconds on, 5 seconds off) suppressed pulsatile LH secretion in ovariectomized (OVX) estradiol (E_2_)-replaced CRH-cre-tdTomato female mice. Infusion of bicuculline (BIC), a GABA_A_ receptor antagonist, or CGP-35348 (CGP), a GABA_B_ receptor antagonist, into the PVN during optical stimulation of PVN CRH neurons differentially modulates LH pulse suppression. Representative LH pulse profiles showing the effects of 20 Hz optical stimulation in CRH-cre mice expressing AAV-ChR2 in the PVN CRH neurons in (A) the absence (OVX) or (B) the presence (OVX + E_2_) of E_2_ replacement. (C) AAV-ChR2 OVX E_2_-replaced mice administered with BIC receiving optical stimulation at 20 Hz. (D) AAV-ChR2 OVX E_2_-replaced mice administered with BIC. (E) AAV-ChR2 OVX E_2_-replaced mice administered with CGP receiving optical stimulation at 20 Hz. (F) AAV-ChR2 OVX E_2_-replaced mice administered with CGP. (G) Control AAV-EYFP mice administered aCSF receiving optical stimulation at 20 Hz. (H) AAV-ChR2 OVX E_2_-replaced mice administered with aCSF in the absence of optical stimulation as a further control. (I) Histogram showing a summary of the mean LH inter-pulse interval for the pretreatment control period (1 hour) and posttreatment period (1 hour) in AAV-ChR2 injected OVX mice (n = 4) receiving optical stimulation; AAV-ChR2 injected OVX + E_2_ mice receiving optical stimulation with (n = 4) or without αCSF infusion (n = 6); AAV-ChR2 injected OVX + E_2_ mice receiving 20 Hz + BIC (n = 6), BIC alone (n = 6), 20 Hz + CGP (n = 6) or CGP alone (n = 6); control virus (AAV-EYFP) injected OVX + E_2_ mice receiving 20 Hz + αCSF (n = 4) and; AAV-ChR2 OVX + E_2_ mice receiving intra-PVN infusion of αCSF in the absence of optical stimulation (n = 3). LH pulses detected by the DynPeak algorithm are indicated with an asterisk. Bolus injections of antagonists or vehicle administered over 10 minutes are indicated by downward black arrows and at the 50-minute time point and were followed by continuous drug infusion at the 60-minute time point indicated by the horizontal black (optical stimulation alone, optical stimulation + antagonist or aCSF) or gray (antagonist or aCSF alone) bar. ^#^*P* < 0.05 vs baseline control period in same treatment group. ^##^*P* < 0.001 vs baseline control period in same treatment group. ^†^*P* < 0.002 vs all other treatment groups. Data represent the mean ± SEM.

After a 1-hour control blood sampling period in the absence of optical stimulation, OVX and E_2_-replaced OVX CRH-cre mice expressing AAV-ChR2-EYFP in PVN CRH neurons, received 1 hour of optical stimulation at 20 Hz. In OVX mice, optogenetic stimulation of PVN CRH neurons at 20 Hz had no effect on the mean LH pulse interval (IPI) (Fig. 4A and 4I, *P* > 0,05, n = 4). However, in E_2_-replaced OVX mice, optical stimulation of PVN CRH neurons with (n = 6) or without αCSF infusion (n = 4) significantly increased mean LH IPI from 16.83 ± 0.95 to 28.75 ± 2.75 minutes (n = 10, mean ± SEM, Fig. 4B and 4I, *P* < .001). There was no significant difference in mean LH IPI during optical stimulation with and without αCSF in E_2_-replaced OVX mice so the data were combined in Fig. 4I (n = 6 and n = 4 respectively, *P* > .05). E_2_-replaced OVX CRH-cre mice also received unilateral infusion of the GABA_A_R antagonist, bicuculline, or the GABA_B_R antagonist, CGP, into the PVN in the presence or absence of 20 Hz of optical stimulation. Administration of BIC into the PVN in the absence of optical stimulation significantly increased the mean LH IPI from 18.73 ± 1.43 to 28.18 ± 4.10 minutes (n = 6, mean ± SEM, *P* < .01, Fig. 4D and 4I). Optogenetic stimulation of PVN CRH neurons during continuous administration of the GABA_A_R antagonist BIC (20 Hz + BIC) profoundly increased the mean LH IPI from 17.78 ± 1.37 to 41.67 ± 4.22 minutes (n = 6, mean ± SEM, Fig. 4C and 4I, *P* < .001). This effect was significantly greater compared with all other groups including optical stimulation (20 Hz) only (*P* < .001) and intra-PVN BIC-only (*P* = .002), indicating the dual treatment of optical stimulation and intra-PVN BIC infusion potentiated the suppressive effects of both treatments. Intra-PVN infusion of the GABA_B_R antagonist CGP during optical stimulation (20 Hz + CGP) blocked the suppressive effect of PVN CRH neuronal activation on LH pulse frequency (n = 5, *P* > .05, Fig. 4E and 4I). Intra-PVN administration of CGP alone did not alter LH pulse frequency (n = 6, *P* > .05, Fig. 4F and 4I). In E_2_-replaced OVX CRH-cre mice induced to express the control viral construct in the PVN, 20 Hz of optical stimulation had no significant effect on LH pulsatility (pretreatment: 18.96 ± 1.57 vs stimulation: 17.71 ± 1.78 minutes; n = 4, mean ± SEM, *P* > .05, Fig. 4G and 4I). Similarly, administration of aCSF in the absence of optical stimulation had no effect on mean LH IPI in OVX E_2_-replaced CRH-cre mice (pretreatment: 19.72 ± 1.69 vs no stimulation: 18.33 ± 1.67 minutes; n = 3, mean ± SEM, *P* > .05, Fig. 4H and 4I).

### Optogenetic Stimulation of PVN GABAergic Projection Terminals in the ARC Suppresses Pulsatile LH Secretion

**Figure 5.**
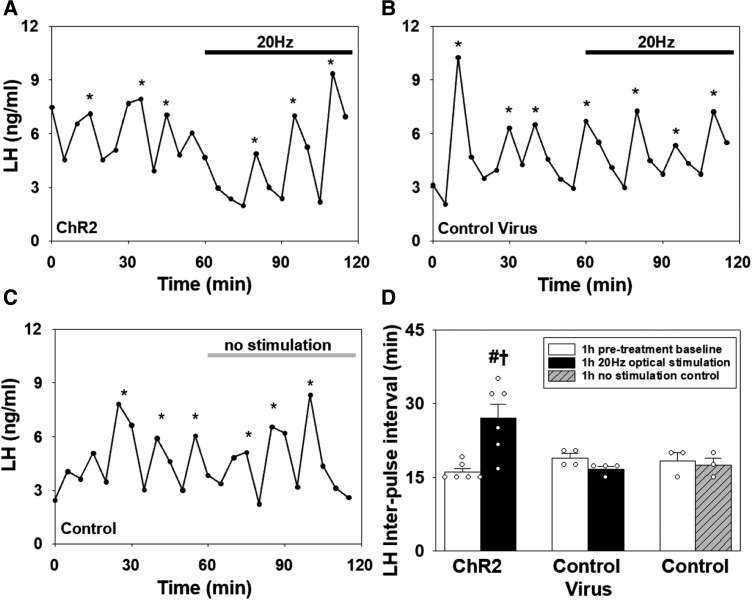
Effects of optogenetic activation of hypothalamic paraventricular nucleus (PVN) GABAergic projection terminals in the hypothalamic arcuate nucleus (ARC) on pulsatile LH secretion in ovariectomized (OVX) estradiol (E_2_)-replaced Vgat-cre-TdTomato female mice. Representative examples showing the effects of (A) AAV-ChR2 injected mice receiving 20 Hz optical stimulation (473 nm, 10 mW, 5 seconds, on 5 seconds off) of PVN GABAergic neuron terminals in the ARC (n = 6), (B) optical stimulation of the ARC in mice injected with a control virus (AAV-EYFP) in the PVN (n = 4), and (C) in the absence of optical stimulation in AAV-ChR2 injected mice (n = 3). (D), Histogram showing a summary of the mean LH inter-pulse interval for all interventions. LH pulses detected by the DynPeak algorithm are indicated with an asterisk. Horizontal black bar indicates period of optical stimulation (473 nm, 20 Hz) and horizontal gray bar indicates absence of light. ^#^*P* < 0.001 vs baseline control period in same treatment group. ^†^*P* < 0.01 vs all other treatment groups. Data represent the mean ± SEM.

After 1-hour blood sampling to determine baseline LH pulse frequency in the absence of optical stimulation, E_2_-replaced OVX Vgat-cre-tdTomato mice expressing AAV-ChR2-EYFP in PVN GABA neurons, received 1 hour of optical stimulation of PVN GABAergic projection terminals in the ARC via a fiber optic cannula implanted in that locus. Optogenetic stimulation at 20 Hz resulted in a significant suppression of pulsatile LH secretion with an increase in mean IPI from 16.11 ± 0.74 to 27.01 ± 2.90 minutes (mean ± SEM; n = 6, *P* < .001, Fig. 5A and 5D). Optical stimulation did not alter LH pulse frequency in E_2_-replaced OVX Vgat-cre-tdTomato mice expressing a control viral construct in the PVN (pretreatment: 18.73 ± 1.03 vs stimulation: 16.58 ± 0.46 minutes; n = 4, mean ± SEM, *P* > .05, Fig. 5B and 5D) or in AAV-ChR2 expressing mice in the absence of optical stimulation (pretreatment: 18.33 ± 1.66 vs no stimulation: 17.50 ± 1.44 minutes; n = 3, mean ± SEM, *P* > .05, Fig. 5C and 5D).

### Activation of PVN CRH Neurons Suppresses LH Pulse Frequency Indirectly Through PVN GABAergic Neurons

**Figure 6.**
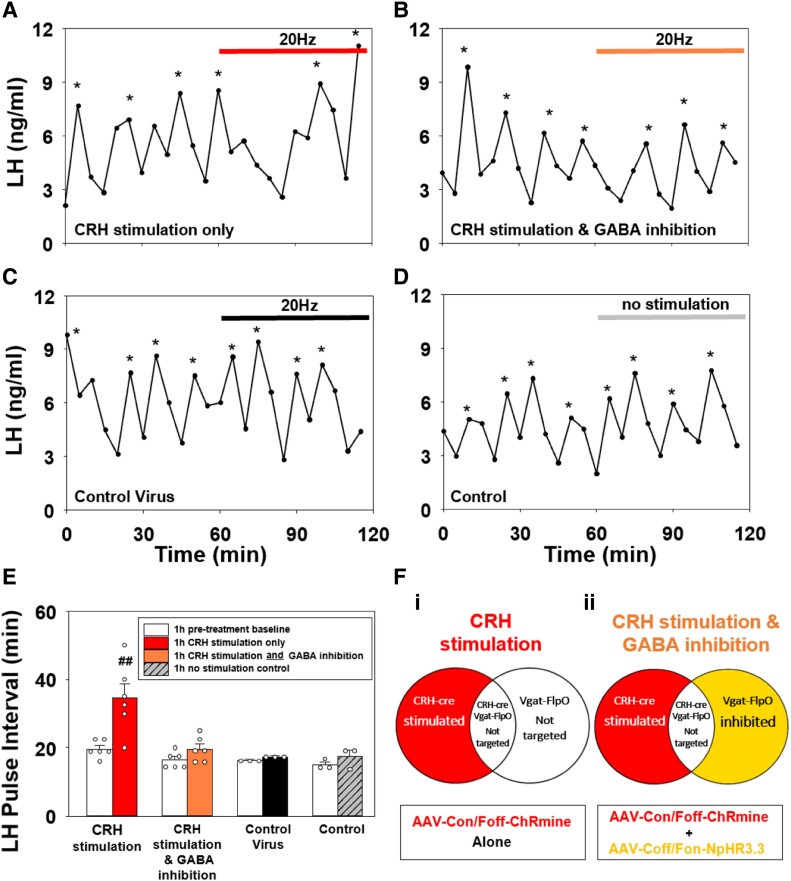
Effect of optogenetic stimulation of non-GABAergic corticotropin-releasing hormone (CRH) neurons with and without simultaneous inhibition of non-CRH GABA neurons in the hypothalamic paraventricular nucleus (PVN), on LH pulse frequency in ovariectomized (OVX) estrogen (E_2_)-replaced female CRH-cre::Vgat-FlpO mice. mice. Optogenetic stimulation (523 nm, 20 Hz, 10 mW, 5 seconds on, 5 seconds off) of non-GABAergic CRH neurons expressing AAV-Con/Foff-ChRmine in the PVN suppressed pulsatile LH secretion in OVX E_2_-replaced CRH-cre::Vgat-FlpO mice. Optogenetic stimulation of PVN non-GABAergic CRH neurons and simultaneous inhibition of non-CRH GABA neurons (523 nm, 20 Hz, 10 mW, 5 seconds on, 5 seconds off) expressing AAV-Con/Foff-ChRmine and AAV-Coff/Fon-NpHR3.3 respectively, did not alter LH pulse frequency in OVX E_2_-replaced CRH-cre::Vgat-FlpO mice. Representative examples showing the effects of (A) 20 Hz stimulation of non-GABAergic CRH neurons (CRH stimulation only) and (B) simultaneous optogenetic stimulation of non-GABAergic CRH neurons and inhibition of non-CRH GABA neurons (CRH stimulation and GABA inhibition). Representative examples showing the effects of (C) optical stimulation in mice expressing control viral constructs and (D) in the absence of optical stimulation in mice expressing AAV-ChRmine. (E) Histograms showing the mean LH inter-pulse interval for the pretreatment control period (1 hour) and posttreatment period (1 hour) for CRH stimulation (n = 6); CRH stimulation and GABA inhibition (n = 6); control virus stimulation (n = 3) and in the absence of stimulation in AAV-ChRmine expressing mice (n = 3). Schematic representing viral construct expression and cells targeted for each group: (Fi) CRH stimulation and (Fii) CRH stimulation and GABA inhibition. LH pulses were determined using the Dynpeak algorithm and are indicated with an asterisk. Horizontal bar in panel (A) indicates period of optical stimulation with 532 nm light (20 Hz) and horizontal bar in panel (B) indicates simultaneous optical stimulation and inhibition with 532 nm light (20 Hz). Horizontal bar in panel (C) indicates period of optical stimulation with 532 nm light (20 Hz) in mice expressing control viral construct, and horizontal bar in panel (D) indicates absence of light for no stimulation control. ^##^*P* < 0.001 vs baseline control period in same treatment group and all other treatment groups. Data represent the mean ± SEM.

We used OVX E_2_-replaced CRH-cre::Vgat-FlpO mice expressing either AAV-Con/Foff-ChRmine only in PVN for selective stimulation of non-GABAergic CRH neurons (Fig. 6Fi) or in combination with AAV-Coff/Fon-NpHR3.3 expression in non-CRH GABAergic neurons for simultaneous stimulation of the CRH neurons and inhibition of the GABAergic neurons (Fig. 6Fii). After a 1-hour blood sampling to determine baseline LH pulse frequency, mice received 1 hour of patterned optogenetic stimulation (20 Hz, 10 mW, 523 nm, 5 seconds on 5 seconds off) to either selectively stimulate PVN CRH neurons alone or simultaneously inhibit GABA neurons only in the PVN. Selective optogenetic stimulation of these non-GABAergic CRH neurons only in the PVN significantly suppressed LH pulse frequency compared to pretreatment baseline (pretreatment: 19.10 ± 1.51 vs stimulation: 33.33 ± 2.79 minutes; n = 6, *P* < .001, Fig. 6A and 6E). Conversely, in CRH-cre::Vgat-FlpO mice expressing both AAV-Con/Foff-ChRmine and AAV-Coff/Fon-NpHR3.3 in CRH and GABAergic neurons, respectively (Fig. 6Fii), LH pulse frequency did not change (pretreatment: 16.39 ± 1.00 vs stimulation + inhibition: 19.99 ± 1.60 minutes; n = 6, mean ± SEM, *P* > .05, Fig. 6B and 6E). Optical stimulation had no effect on LH pulse frequency in mice expressing a mixture of control viral constructs (pretreatment: 16.11 ± 0.56 vs stimulation: 17.22 ± 0.55 minutes; n = 3, mean ± SEM, *P* > .05, Fig 6C and 6E) or in mice expressing AAV-ChRmine alone that did not receive optical stimulation (pretreatment: 15.00 ± 0.96 vs poststimulation: 17.36 ± 1.87 minutes; n = 3, mean ± SEM, *P* > .05, Fig. 6D and 6E).

### Optical Stimulation of PVN CRH Neurons Increases CORT Secretion in OVX and E_2_-replaced Mice

**Figure 7.**
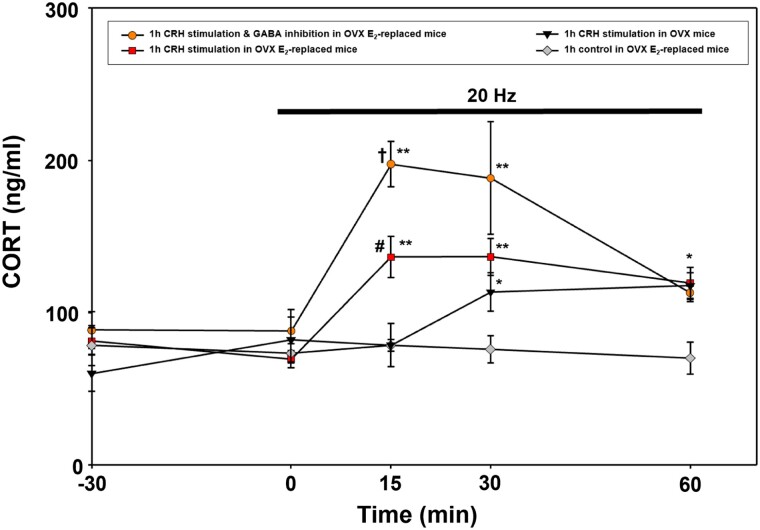
Effect of optogenetic stimulation of corticotropin-releasing hormone (CRH) neurons in the hypothalamic paraventricular nucleus (PVN) in the presence or absence of simultaneous inhibition of non-CRH GABA neurons on corticosterone (CORT) secretion in ovariectomized (OVX) estrogen (E_2_)-replaced CRH-cre and CRH-cre::Vgat-FlpO mice. CORT secretion time-course for the response to optical stimulation (20 Hz, 10 mW, 5 seconds on, 5 seconds off) of PVN CRH neurons in OVX mice (AAV-ChR2, n = 4, triangles); OVX E_2_-replaced mice receiving optical stimulation of CRH neurons only (AAV-ChR2, n = 4 ; AAV-Con/Foff-ChRmine, n = 6; combined as squares) and in OVX E_2_-replaced mice receiving optical stimulation of CRH neurons and simultaneous inhibition of PVN GABA neurons (AAV-Con/Foff-ChRmine and AAV-Coff/Fon-NpHR3.3, n = 5, circles), and OVX E_2_-replaced control group (AAV-EYFP/mCherry, 20 Hz stimulation, n = 3; AAV-ChR2, no stimulation n = 3; combined as diamonds). Optical stimulation was initiated at time point 0 minutes and terminated at 60 minutes (1 hour duration). **P < 0.001 vs time points −30 and 0 minutes in the same treatment group; *P* < 0.006 vs OVX E_2_-replaced control at same time point. *P < 0.05 vs time points −30 and 0 minutes in the same treatment group; OVX E_2_-replaced control at same time point. ^†^*P* < 0.05 vs CRH stimulation in OVX E_2_-replaced mice at same time point; *P* < 0.001 vs CRH stimulation in OVX mice at same time point. ^#^*P* < 0.05 vs CRH stimulation in OVX at same time point. Data represent the mean ± SEM.

Optogenetic stimulation (AAV-ChR2, 473 nm, 20 Hz) of PVN CRH cell bodies in OVX mice elevated CORT secretion at time points 30 and 60 minutes compared with pretreatment baseline and controls (*P* < .05, n = 4, Fig. 7). Optogenetic stimulation of PVN CRH cell bodies in OVX E_2_-replaced mice elevated CORT secretion compared with pretreatment baseline and controls at time point 15, 30, and 60 minutes (combining AAV-ChR2, n = 4 and AAV-Con/Foff-ChRmine, n = 6, *P* < .05, Fig. 7). Furthermore, optical stimulation of PVN CRH cell bodies in OVX E_2_-replaced mice induces a more rapid increase in CORT secretion compared with OVX mice which was evident at time point 15 minutes (*P* < .05). Optogenetic stimulation of PVN CRH neurons in combination with simultaneous inhibition of PVN GABA cell bodies resulted in significantly greater increase in CORT secretion (AAV-Con/Foff-ChRmine and AAV-CoffFon-NpHR3.3, 532 nm, 20 Hz, n = 5, *P* < .05, Fig. 7) compared with CRH stimulation alone at time points 15 and 30, but this CORT response was not sustained and returned to levels equivalent to CRH stimulation alone at 60 minutes (*P* < .05, Fig. 7). There was no significant change in circulating CORT levels in OVX E_2_-replaced control animals (control virus, AAV-YFP, and AAV-mCherry, optically stimulated at 20 Hz [n = 3] or AAV-ChR2 with no optical stimulation [n = 3], which were therefore combined in Fig. 7).

## Discussion

The present study shows that optogenetic stimulation of PVN CRH neurons is only effective at suppressing LH pulse frequency in OVX mice with E_2_ replacement and not in its absence. These results reaffirm the role of PVN CRH neurons as modulators of LH pulse frequency and demonstrate that circulating E_2_ plays a permissive role in enabling LH pulse suppression during PVN CRH neuronal activation. In rodents, gonadal steroids increase PVN CRH mRNA expression (37) and PVN CRH mRNA levels and CRH neuronal excitability peak during the proestrous stage of the estrous cycle when E_2_ levels are highest (38, 39). The amygdala and bed nucleus of the stria terminalis (BNST) express estrogen receptor (ER) in GABAergic and glutamatergic neurons (40) and are known to activate the HPA axis by providing disinhibitory input to the PVN (11). Therefore, it is possible E_2_ may be acting upstream of the PVN in ER expressing brain regions to alter inhibitory and excitatory tone to the PVN. However, there is evidence to suggest the PVN per se is the site of the sensitizing effects of E_2_ on PVN CRH-mediated LH pulse suppression. Intra-PVN administration of E_2_ results in an immediate suppression of LH pulse frequency in stressed, but not in unstressed, conditions (19) and an increase in CORT secretion under basal conditions (41). The immediate effects of intra-PVN infusion of E_2_ on neuroendocrine function suggests a nongenomic mechanism may also underpin the permissive effects of E_2_ on stress-induced suppression of LH pulse frequency. CRH neurons in the PVN have been shown to express the membrane-bound ERs, GPR30 and Gq-mER (42, 43). Electrophysiological recordings from PVN CRH neurons in vitro show that application of a selective Gq-mER agonist increases their excitability and firing rate (43). Therefore, it is possible that E_2_ may be acting directly on CRH neurons in the PVN and that their increased excitability enhances their responsivity to optogenetic stimulation or stress related inputs and consequently enable LH pulse suppression. In rodents, PVN CRH neurons mostly colocalize with ER-β and not ER-α (44, 45). Conversely, GABA neurons within the PVN and surrounding peri-PVN region colocalize with ER-α (46, 47). Administration of a selective agonist for ER-α, but not ER-β, results in an increase of CORT secretion in basal conditions and potentiates CORT secretion during stress exposure (41). Therefore, the permissive effects of E_2_ on pulsatile LH secretion during optogenetic activation of CRH neurons may also be linked to a reduction in inhibitory GABAergic regulation of PVN CRH neurons, particularly because E_2_ has been shown to alter GABA receptor function by decreasing GABA_A_R density at synaptic sites (48). Furthermore, local CRH type 1 receptor (CRHR1) expressing neurons in the PVN have been shown to colocalize with ER-α (49) and are also implicated in modulating PVN CRH neuronal activity via synaptic negative feedback (10); therefore, E_2_-dependent changes in intra-PVN GABA signalling may also contribute to enhanced CRH neuronal activation during optical stimulation.

Increased CORT secretion has also been associated with suppressed reproductive function in an E_2_-dependent manner (6). Because stimulating PVN CRH neurons increased CORT secretion in both OVX and OVX E_2_-replaced mice, but to a greater degree in the latter, it is possible that CORT per se may be acting directly on KNDy neurons (5, 6) to suppress LH pulse frequency. However, acute CORT administration in OVX E_2_-replaced female rats and mice does not alter LH pulse frequency or amplitude (2, 50), suggesting that the increased CORT secretion induced in the present study would not affect LH pulse frequency.

Although the present study confirms that unilateral manipulation of PVN CRH neurons is sufficient to suppress the GnRH pulse generator, it is limited in that it did not examine the effects of bilateral stimulation of PVN CRH neurons, which would be physiologically ideal. Interestingly, it was recently shown that selective chemogenetic bilateral activation of PVN CRH neurons resulted in a profound suppression of pulsatile LH secretion in OVX mice without the need for E_2_ replacement (21). This might be explained by the different methodology used, in particular the bilateral activation of the PVN CRH neuronal population or the manipulation of Gq-mediated signalling during DREADD-mediated activation of PVN CRH neurons (21) compared with the unilateral optogenetic targeting used in the present study. The present finding that E_2_ replacement is necessary for LH pulse suppression during optical stimulation of PVN CRH neurons is consistent with previous reports that E_2_ has a sensitizing effect on CRH-mediated LH pulse suppression (37, 51). Central administration of CRH only suppressed LH pulse frequency in E_2_-replaced OVX rats and not in the absence of the steroid (37).

**Figure 8.**
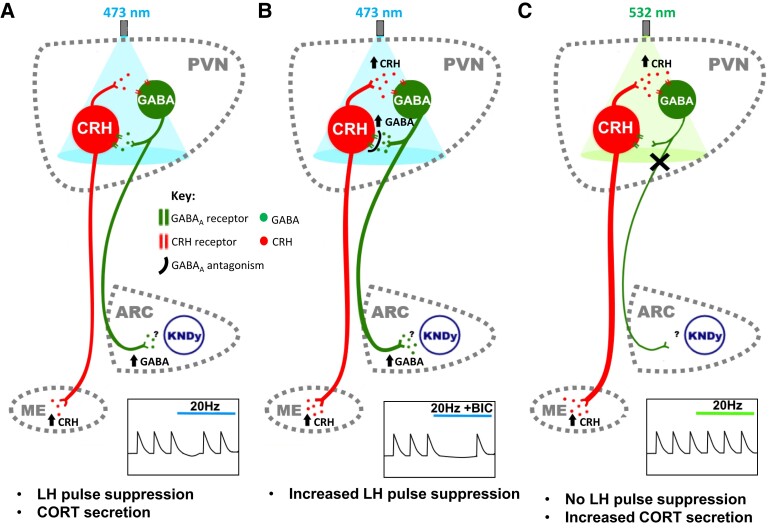
Schematic illustration of proposed model of hypothalamic paraventricular nucleus (PVN) neurocircuit. (A) Optogenetic stimulation of PVN corticotropin-releasing hormone (CRH) neurons releases CRH locally, which activates local CRH receptor expressing GABA neurons that in turn project to the arcuate (ARC) KNDy network to increase inhibitory tone and decrease GnRH pulse generator frequency. (B) GABA_A_ antagonism during optical stimulation of PVN CRH neurons blocks the local negative feedback onto PVN CRH neurons resulting in greater activation of ARC projecting GABA neurons, and consequently, potentiated LH pulse suppression. (C), Simultaneous inhibition of local PVN GABA neurons during optical stimulation of PVN CRH neurons prevents CRH-mediated suppression of the GnRH pulse generator despite an increase in corticosterone (CORT) secretion. ARC, arcuate nucleus; KNDy, kisspeptin neurones colocalizing neurokinin-B and dynorphin; ME, median eminence; PVN, paraventricular nucleus.

The present study shows that GABA signalling in the PVN plays opposing roles in mediating LH pulse suppression in response to PVN CRH optogenetic activation, which is dependent on GABA receptor subtypes. Administration of GABA_A_R antagonist greatly potentiated, whereas GABA_B_R antagonist reversed, LH pulse suppression induced by optogenetic activation of PVN CRH neurons in OVX E_2_-replaced female mice. Neuronal activation of PVN CRH neurons is tightly regulated by GABAergic signalling with more than a third of synaptic inputs to PVN CRH neurons identified as GABAergic (52). Tract tracing studies show inputs from the limbic system, hypothalamic nuclei, the peri-PVN region, and locally from within the PVN to PVN CRH neurons (53). Both GABA_A_ and GABA_B_ receptors play differential roles in regulating PVN CRH neurons, with GABA_A_R signalling constrains PVN CRH neuronal activity by regulating rapid phasic and persistent tonic inhibition occurring at synaptic and extrasynaptic sites on CRH neurons, respectively (15). Whereas GABA_B_R signalling has been shown to increase CRH firing rate by presynaptically binding to GABA_B_R to decrease GABA release and thereby reducing GABA_A_-mediated inhibition of CRH neurons (15). The administration of GABA_A_R antagonist into the PVN may be acting to enhance CRH-mediated suppression of LH secretion by blocking GABA inhibition of CRH neurons resulting in increased CRH transmission. Conversely, administration of GABA_B_R antagonist may be blunting LH pulse suppression during PVN CRH neuronal activation by blocking autoinhibition of GABA release from presynaptic terminals. GABAergic signalling within the PVN plays a key role in mediating HPA axis activation during stress exposure and increased GABAergic output from limbic system nuclei is believed to disinhibit PVN CRH neurons to induce CRH and consequently CORT release (11). There is also evidence to suggest that GABA_A_R-mediated inhibition of PVN CRH neurons is absent during stress. Infusion of a GABA_A_R antagonist into the PVN results in an increase in CORT secretion in unstressed rats; however, this did not further increase raised CORT levels in stressed animals (54). A recent study using mathematical and in vivo electrophysiological approaches strongly suggests that recurrent inhibitory networks maintain low-activity firing dynamics in CRH neurons and predicts that disinhibition of CRH neurons shifts firing dynamics into a high-activity state (55). In the present study, the substantially stronger LH pulse suppression observed with optical stimulation of CRH neurons combined with GABA_A_R antagonists may be mirroring the loss of recurrent inhibition resulting in increased CRH release (Fig. 8A and 8B). Acute stress has been shown to remove synaptic inhibition to PVN CRH neurons via a depolarizing shift in GABA_A_ signalling (54).

Although the present findings confirm previous reports that PVN CRH neurons are modulators of LH pulse frequency (21), the neural circuit mediating LH pulse suppression following PVN CRH neuronal activation remains poorly understood. The PVN has been identified as a major source of afferents to ARC KNDy neurons, which may suggest a direct CRH projection from the PVN to the GnRH pulse generator, particularly because ARC kisspeptin neurons have been shown to express CRH receptors (23). However, primary afferents from the PVN to the KNDy network were initially reported to not express CRH (22). Although more recent reports have confirmed close appositions between PVN CRH terminals and ARC kisspeptin neurons, brain-slice electrophysiology studies have demonstrated that both direct application of CRH and optogenetic stimulation of PVN CRH terminals in the ARC were ineffective at altering ARC kisspeptin firing rate (21), suggesting an indirect mechanism mediates the suppressive effects of increased PVN CRH neuronal activity on the GnRH pulse generator.

Recently, a local circuit between CRH and GABAergic CRH-R1 expressing neurons in the PVN has been described, which plays an essential role in regulating CRH neuronal activity through synaptic negative feedback (10). These CRH-responsive GABA neurons have been shown to make local intra-PVN connections as well as long-range projections to extrahypothalamic nuclei (10). It is possible that activation of PVN CRH neurons suppresses GnRH pulse generator activity by stimulating these CRH-responsive GABA projection neurons, particularly because the PVN provides a major source of direct innervation to the KNDy network, although the neurochemical phenotype of these projections remains undetermined (22, 56). In the present study, we determined if a functional GABA projection from the PVN to the ARC exists and is capable of influencing LH pulse frequency. Optogenetic stimulation of PVN GABA projection terminals in the ARC suppresses LH pulse frequency in Vgat-cre OVX E_2_-replaced female mice. These data confirm a functionally relevant GABAergic projection from the PVN to the ARC, the site of the GnRH pulse generator. Although it is possible that this PVN GABA projection directly synapses onto ARC KNDy neurons, especially because monosynaptic tract tracing studies have confirmed direct communication between the PVN and KNDy network (22, 56), and we observed PVN GABA projection fibers in the ARC, we have not confirmed the phenotypic identity of the neurons they may be communicating with, which is a limitation of this study and requires further experimentation. Therefore, we cannot exclude the possibility of an indirect communication between PVN GABA terminals in the ARC and the KNDy network.

Although PVN CRH neurons are predominantly glutamatergic (57), early immunochemistry (58) and more recent single-cell RNA sequencing (59, 60) studies have suggested that a subset of PVN CRH neurons may express GABA. To confirm the phenotype of CRH neurons mediating LH pulse suppression, we used intersectional strategies to selectively target and stimulate CRH neurons that do not express GABA. Optogenetic stimulation of non-GABAergic CRH cell bodies in the PVN still resulted in suppression of pulsatile LH secretion (Fig. 8A). Therefore, our present finding in this study of a novel functional PVN to ARC GABAergic projection is unlikely to coexpress CRH suggesting an indirect mechanism whereby PVN CRH neurons stimulate a GABA projection to the ARC to alter GnRH pulse generator frequency. This is consistent with previous findings that optogenetic stimulation of PVN CRH terminals in the ARC did not alter kisspeptin firing, indicating an indirect mechanism (21). To confirm if PVN CRH neurons suppress pulsatile LH secretion through PVN GABA neurons, we selectively stimulated non-GABAergic CRH neurons while simultaneously inhibiting PVN GABA neurons that do not express CRH. We found that inhibition of these PVN GABA neurons completely blocked LH pulse suppression mediated by activation of PVN CRH neurons (Fig. 8C). These findings strongly suggest that CRH neurons in the PVN suppress GnRH pulse generator activity by signalling through a local PVN GABA population. Furthermore, we found that simultaneous inhibition of PVN GABA neurons during optical stimulation of PVN CRH neurons induced a significantly greater elevation of CORT compared with CRH stimulation alone. This finding is consistent with previous studies whereby selective ablation of predominantly GABAergic CRH-R1 containing neurons in the PVN resulted in increased CORT secretion in response to immobilization stress (10). Because a plethora of evidence suggests that the loss of synaptic inhibition to PVN CRH neurons increases CRH neuronal activity (11, 54, 55), inhibition of PVN GABA neurons may be increasing CORT secretion via disinhibition of CRH neurons leading to increased CRH signalling in response to optical stimulation. Therefore, the finding that inhibition of PVN GABA neurons completely blocked suppression of pulsatile LH secretion despite increased CRH activity and CORT secretion provides even more compelling evidence for an indirect mechanism whereby PVN CRH neurons stimulates PVN GABA neurons, which in turn inhibits GnRH pulse generator activity. Although we show a clear functional GABAergic projection from the PVN to the ARC, and that inhibition of PVN GABA neurons blocks CRH-mediated suppression of the GnRH pulse generator, it remains to be determined if this is due to the direct activation of this ARC projection. PVN CRH-R1 GABA neurons make long-range connections to different higher order brain nuclei such as the BNST and brainstem nuclei including the locus coeruleus (10). Therefore, we cannot exclude the possibility of alternative pathways involving PVN GABA projections to nuclei other than the ARC, particularly because both the BNST (61) and the locus coeruleus (51) have been implicated in mediating stress-induced suppression of LH pulse frequency. CRHR1 neurons in the PVN also receive inputs from the BNST and brain stem nuclei (10); therefore, bidirectional communication between these brain regions may be involved in mediating stress-induced suppression of reproductive function. Indeed, as the apex of the HPA axis, the PVN is a major site of convergence of information regarding stress-related stimuli and may therefore be a candidate site for integrating the stress and reproductive axes.

The neural circuit outlined in the present study (Fig. 8) may also play a role in mediating stress-induced suppression of the GnRH pulse generator. However, a limitation of the present study is that we have not confirmed whether the PVN GABA neurons targeted are activated during optical stimulation of the PVN CRH neurons. Nevertheless, optical stimulation of PVN CRH neurons has previously been shown to increase the firing rate of local CRHR1 neurons, of which 90% are GABAergic (10, 62). Therefore, it is likely that the optical stimulation of PVN CRH neurons also activates local GABA neurons. PVN GABA neurons may also be stress responsive because they display increased neuronal activation during acute and chronic social defeat stress (62). Transsynaptic tracing experiments suggest that metabolic signals detected by the hindbrain may be relayed to ARC KNDy neurons via the PVN (63). Furthermore, lesions of the PVN partially block fasting-induced suppression of the LH secretion in male rats (17) and increased adrenergic input to the PVN, presumably from the brainstem (64), has been shown to mediate metabolic stress-induced suppression of the GnRH pulse generator through CRH signalling (65). Many brain regions associated with mediating stress-induced suppression of the GnRH pulse generator such as the posterodorsal subnucleus of the medial amygdala (MePD) (36, 66), BNST (61) and the locus coeruleus (51) provide direct synaptic innervation to PVN CRH neurons (67), consistent with their role in modulating HPA axis activity. Urocortin-3 (UCN3) and GABA signalling in the MePD has been implicated in mediating psychogenic stress-induced suppression of the GnRH pulse generator (36, 66). Recently, we have shown that selective stimulation of MePD UCN3 terminals in the PVN suppresses LH pulse frequency and increases CORT secretion in OVX E_2_-replaced mice (68). PVN CRH neurons have been shown to express CRHR2 (57), for which UCN3 is a selective ligand; therefore, it is possible that MePD UCN3 terminals in the PVN may be modulating LH pulsatility through PVN CRH neurons. However, studies examining the role of the MePD in stress-induced suppression of LH pulse frequency were conducted in OVX only mice and a direct GABAergic projection from the MePD to the ARC, which modulates LH pulse frequency has also been described (66). Therefore, direct, and E_2_-independent pathways not involving the PVN are very likely to also relay stress cues to the reproductive axis. The presence of multiple pathways mediating stress-induced suppression of reproductive function may also explain why early lesion studies of the PVN found that the PVN was not essential for mediating the effects of different stressors on pulsatile LH secretion (16). Redundancy is the necessary basis of robustness. Further work will be needed to verify the physiological significance of our proposed circuit in regulating stress-induced suppression of the GnRH pulse generator.

In the present study, we found that optical stimulation of PVN CRH neurons or PVN GABA terminals in the ARC induced a transient suppression of the GnRH pulse generator despite ongoing stimulation. This contrasts with our previous findings that optical stimulation of amygdala GABA projection terminals in the ARC resulted in sustained suppression of LH pulses for the duration of optical stimulation (66). As suggested in the schematic neural circuit in Fig. 8, GABAergic inhibitory synaptic feedback onto PVN CRH neurons provides a mechanism that may limit the stress response and promote stress recovery. By optically stimulating PVN CRH neurons, the resulting activation of local CRHR1 GABA neurons both suppresses the GnRH pulse generator but also feeds back to exert an inhibitory tone onto PVN CRH neurons. Therefore, this increased GABAergic feedback may decrease the excitability of CRH neurons and consequently result in a transient suppression of LH pulse frequency. Interestingly, when we administered a GABA_A_R antagonist into the PVN during optical stimulation of the CRH neurons we induced a sustained suppression of LH pulse frequency, which may have resulted from blocking the synaptic negative feedback onto the CRH neurons leading to a greater activation of GABA projections to the ARC KNDy network. Similarly, optical stimulation of PVN GABA terminals in the ARC produced a transient suppression of LH pulse frequency, which may also be a result of synaptic feedback activity within the PVN. Optogenetic stimulation of projection terminals may induce retrograde propagation of action potential, which could activate collateral branches. In this instance, back propagation of action potentials may result in increased local GABA release in the PVN, inhibiting CRH neurons in the PVN and consequently decreasing PVN GABA neuron activity (10). The engagement of this GABAergic synaptic feedback mechanism in the PVN may therefore produce transient inhibition of the GnRH pulse generator despite sustained optical stimulation of the PVN CRH neurons. This is supported by the finding that inhibition of local GABA neurons during optical stimulation of PVN CRH neurons also resulted in increased CORT secretion compared with CRH stimulation alone. Inhibition of the GABAergic synaptic feedback mechanism is likely resulting in greater activation of PVN CRH neurons and CRH release at the level of the median eminence.

In summary, the present study reaffirms the role of PVN CRH neurons as modulators of LH pulse frequency and demonstrates a potential sensitizing role of E_2_ in CRH-mediated LH pulse suppression. Furthermore, we show for the first time that GABA signalling in the PVN differentially modulates LH pulse frequency in response to optogenetic activation of PVN CRH neurons. Based on the novel intersectional and optogenetic strategy used in the present studies, we show that PVN CRH neurons mediate LH pulse suppression through GABAergic signalling intrinsic to the PVN that may incorporate a functionally significant PVN GABAergic projection to the hypothalamic GnRH pulse generator.

## Data Availability

Some or all datasets generated during and/or analyzed during the current study are not publicly available but are available from the corresponding author on reasonable request.

## Abbreviations

aCSF: artificial cerebrospinal fluid
ARC: arcuate nucleus
BIC: bicuculline
BNST: bed nucleus of the stria terminalis
CORT: corticosterone
CRH: corticotropin-releasing hormone
CRHR1: corticotropin-releasing hormone type 1 receptor
E_2_: estrogen
ER: estrogen receptor
HPA: hypothalamus-pituitary-adrenal
IPI: inter-pulse interval
KNDy: kisspeptin/neurokinin B/dynorphin
MePD: posterodorsal subnucleus of the medial amygdala
OVX: ovariectomized
PVN: paraventricular nucleus
UCN3: urocortin-3
